# Exploring the vaccine-induced immunity against severe acute respiratory syndrome coronavirus 2 in healthcare workers

**DOI:** 10.1038/s41598-023-33397-4

**Published:** 2023-04-26

**Authors:** Yong Kwan Lim, Oh Joo Kweon, Yoojeong Choi, Sumi Yoon, Tae-Hyoung Kim, Mi-Kyung Lee

**Affiliations:** 1 Department of Laboratory Medicine, Chung-Ang University College of Medicine, 102 Heukseok-ro, Dongjak-gu, Seoul, 06973 South Korea; 2 Department of Urology, Chung-Ang University College of Medicine, Seoul, South Korea

**Keywords:** Infectious diseases, Innate immune cells, Infectious diseases

## Abstract

We aimed to analyze the kinetics of T-cell-mediated and B-cell-mediated humoral immune responses against severe acute respiratory syndrome coronavirus 2 (SARS-CoV-2) before and after booster vaccination, as well as the impacts of the in vitro test results the type of vaccination on the prediction of SARS-CoV-2 infection. A total of 240 healthcare workers vaccinated twice were serially tested using an interferon gamma release assay (IGRA) and a neutralizing antibody (nAb). At the end of the study, we investigated the history of SARS-CoV-2 infection of all the enrolled participants to analyze the effects of the test results and the type of vaccination on SARS-CoV-2 infection. Overall, the positive rates were 52.3% and 80.0% for IGRA and 84.6% and 100% for the nAb test before and after booster vaccination, respectively. However, the positive rates were 52.8% for IGRA and 100% for nAb 3 months after booster vaccination. The in vitro test results and the type of vaccination were not associated with SARS-CoV-2 infection. The antibody response caused by the SARS-CoV-2 vaccination lasted more than 6 months, although the response of the T-cells disappeared rapidly after 3 months. However, these in vitro results and the type of vaccination cannot be used for predicting the risk of SARS-CoV-2 infection.

## Introduction

As of 19 February 2023, COVID-19 led to 6.9 million deaths globally due to the rapid spread of Severe Acute Respiratory Syndrome Coronavirus 2 (SARS-CoV-2)^1^. With the unprecedented, rapid spread of this novel respiratory disease, there was a global race to discover and deploy a vaccine against COVID-19 to achieve herd immunity^2^. Due to these efforts, several vaccines had been validated by WHO, which showed various efficacies such as preventing the spread of the virus and decreasing the risk of serious health consequences^3^.

As of 12 December 2022, approximately 13 billion vaccine doses have been administered worldwide^4^. With this large scale of vaccination, the public interest in immunity against COVID-19 has been growing. Currently, in vitro methods to evaluate the immune status against pathogens consist of two pivotal axes: the neutralizing antibody (nAb) test for the evaluation of B-cell-mediated humoral response and the interferon-γ (INF-γ) release assay (IGRA) against the SARS-CoV-2 antigen for detecting the T-cell mediated response. The clinical performance of these assays has been globally evaluated, and the IGRA format assay is useful for predicting the severity of infection and clinical prognosis for COVID-19 patients and evaluating T-cell immune response following COVID-19 vaccination^5–8^. Similarly, the nAb test showed acceptable performance for the evaluation of B-cell immune status against COVID-19 in clinical settings^9,10^. However, how the results of these tests vary according to the type of vaccination is still unclear. In addition, there is very little information available regarding the association between the test results and SARS-CoV-2 infection over a long-term follow-up period.

In this study, we enrolled healthcare workers (HCWs), who received two doses of a COVID-19 vaccine and planned to receive booster vaccination, and performed the QuantiFERON-TB SARS-CoV-2 ELISA Kit (QFN-SARS; QIAGEN, MD, USA) and the ichroma™ COVID-19 nAb assay (ichroma nAb; Boditech Med, Gangwon-do, Republic of Korea) for all participants. With these test results, we analyzed the kinetics of the IGRA and nAb test results with respect to the type of the first and second vaccinations and the number of elapsed days after the last vaccination. Additionally, at the end of the study period, we checked the COVID-19 infection history of all the participants and analyzed the impacts of the in vitro test results and the type of vaccination on the prediction of SARS-CoV-2 infection.

## Results

A total of 293 specimens from 240 HCWs were tested using QFN-SARS and ichroma nAb assays. These tests were conducted in November 2021 and February 2022. Of all enrolled HCWs, 65 HCWs did not receive the booster vaccination during the study period (booster (−) group), and 175 participants were vaccinated with a booster shot (booster (+) group).

The basic characteristics of the participants are summarized in Table 1. For the HCWs of the booster (−) and booster (+) groups, the median ages (years) were 36 and 43, and the proportions of female HCWs were 66.2% and 70.9%, respectively. The first and second doses were of the following types of vaccines: AstraZeneca COVID-19 Vaccine (AZ), Pfizer-BioNTech COVID-19 vaccine (PF), or Moderna COVID-19 Vaccine (MO). PF was the only vaccine used as the booster shot. There were four combinations of the first and second vaccines for the booster (−) and booster (+) groups: first and second doses of AZ (1AZ–2AZ), first dose of AZ and second dose of PF (1AZ–2PF), first and second doses of PF (1PF–2PF), and first and second doses of MO (1MO–2MO). At the end of the study, we retrospectively checked all participants for COVID-19 infection; 33.8% (booster (−) group) and 41.1% (booster (+) group) of the HCWs had been infected with SARS-CoV-2.

**Table 1:** Basic characteristics of enrolled healthcare workers (HCWs).

	Booster (−) group (*n* = 65)		Booster (+) group (*n* = 175)	
Age	36	(30–46)	43	(37–50)
Female	43	(66.2%)	124	(70.9%)
Type of vaccination				
1AZ–2AZ	22	(33.8%)	117	(66.9%)
1AZ–2PF	7	(10.8%)	23	(13.1%)
1MO–2MO	14	(21.5%)	1	(0.6%)
1PF–2PF	22	(33.8%)	34	(19.4%)
1st blood sampling				
Number of tested HCWs	65		175	
Days between last vaccine dose and 1st test	141	(94–204)	20	(19–22)
2nd blood sampling				
Number of tested HCWs	N/A		53	
Days between last vaccine dose and 2nd test	N/A		85	(84–87)
COVID-19 infection history	22	(33.8%)	72	(41.1%)

Data are shown as median with interquartile range or number with percentage.

*AZ* AstraZeneca COVID-19 Vaccine, *MO* Moderna COVID-19 Vaccine, *N/A* not applicable, *PF* Pfizer-BioNTech COVID-19 vaccine.

**Figure 1:**
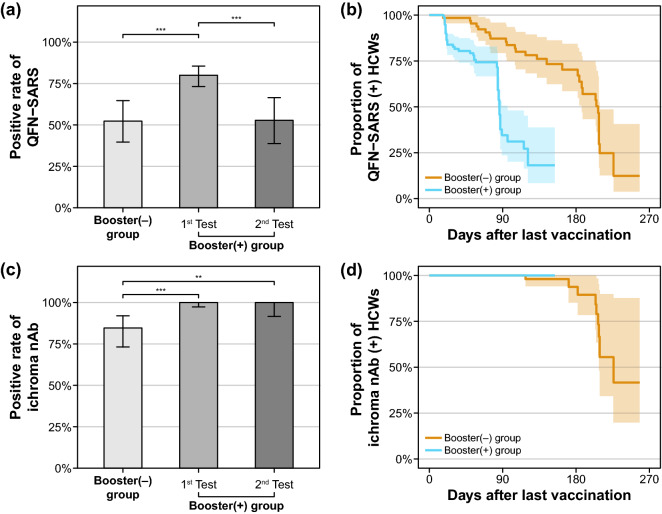
Positive rates of QFN-SARS (**a**) and ichroma nAb (**c**) before and after booster shot. Asterisks indicate significant differences (**p < 0.01, ***p < 0.001), and error bars indicate 95% confidence intervals of the positive rates. Kaplan–Meier curves displaying the proportion of QFN-SARS-positive (**b**) and ichroma nAb-positive (**d**) HCWs with respect to the days elapsed after the final vaccine dose.

**Figure 2:**
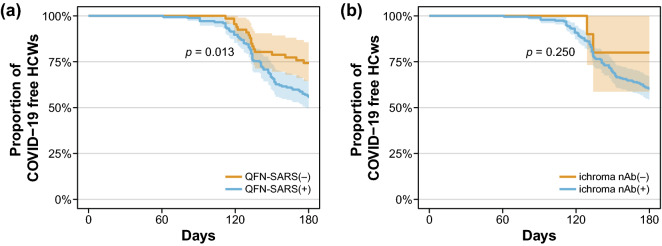
Kaplan–Meier plots of COVID-19-free follow-up days based on initial QFN-SARS results (**a**) and ichroma nAb results (**b**) for all enrolled HCWs.

The first blood sampling was performed at a median of 141 days for the booster (−) group and 20 days for the booster (+) group after the last vaccine dose. The positive rates in the first QFN-SARS and ichroma nAb tests were 52.3% and 84.6% for the booster (−) group and 80.0% and 100% for the booster (+) group, respectively (Fig. 1). After 3 months, the second test was performed in some participants of the booster (+) group (*n* = 53), and the positive rates of QFN-SARS and ichroma nAb were 52.8% and 100%, respectively. In QFN-SARS results, the positive rate was statistically high in the 1st test of booster (+) group (*p* < 0.001) compared to the booster (−) group and the 2nd test of booster (+) group, and in ichroma nAb results, the positive rate was significantly low in the booster (−) group (*p* < 0.001) compared to the 1st and 2nd tests of booster (=) group. When considering only the QFN-SARS result of 53 HCWs who had tested both 1st and 2nd tests, the positive rates were 90.6% (48/53) and 52.8% (28/53) for 1st test and 2nd test, respectively (*p* < 0.001). Additionally, with the first and second test results from all participants, we performed Kaplan–Meier analyses to analyze the kinetics of QFN-SARS and ichroma nAb results depending on the number of days after the last vaccine dose. The proportion of QFN-SARS-positive participants showed a tendency to drop gradually, and approximately half of the booster (+) and booster (−) HCWs were QNF-SARS-negative after 3 and 6 months following the last vaccination, respectively. For ichroma nAb, the nAb-positive participants also showed a declining trend as time elapsed after the last vaccine dose; however, approximately 80% of the booster (−) participants had nAb against SARS-CoV-2 until 6 months following the last dose. The positive rate of nAb was 100% in booster (+) group at the end of the study period. In addition, the time from the initial test to COVID-19 diagnosis was estimated utilizing Kaplan–Meier analysis with the endpoint of time-to-COVID-19-infection (Fig. 2). When comparing the QFN-SARS-positive and -negative groups, the proportion of COVID-19-free HCWs during the study period was significantly higher among the QFN-SARS-negative participants. Similarly, ichroma nAb-negative participants were less likely (but not significantly) to be infected with SARS-CoV-2.

Table 2 shows the results of QFN-SARS and ichroma nAb together with COVID-19 infection history based on the type of SARS-CoV-2 vaccination. In the booster (−) group, the positive rates of QFN-SARS in the 1MO–2MO group and of ichroma nAb in the 1AZ–2PF, and 1MO–2MO groups tend to have a higher positive rates. In the booster (+) group, there was no significant difference in the positive rates of QFN-SARS for the 1st and 2nd tests with respect to the type of vaccination, and all of them were positive for ichroma nAb. In addition, we could not determine any difference in the COVID-19 infection rates according to the vaccine type in the booster (−) and booster (+) groups.

**Table 2:** The results of QFN-SARS and ichroma nAb and infection history of COVID-19 with respect to the type of vaccination.

	1st test result				2nd test result				COVID-19 infection history	
	QFN-SARS		ichroma nAb		QFN-SARS		ichroma nAb			
Booster (−) group (n = 65)										
1AZ–2AZ	7/22	(31.8%)	15/22	(68.2%)	N/A		N/A		6/22	(27.3%)
1AZ–2PF	4/7	(57.1%)	7/7	(100%)	N/A		N/A		1/7	(14.3%)
1MO–2MO	12/14	(85.7%)	14/14	(100%)	N/A		N/A		6/14	(42.9%)
1PF–2PF	11/22	(50.0%)	19/22	(86.4%)	N/A		N/A		9/22	(40.9%)
* p*-value	0.018		0.038						0.454	
Booster (+) group (n = 175)										
1AZ–2AZ	93/117	(79.5%)	117/117	(100%)	18/38	(47.4%)	38/38	(100%)	48/117	(41.0%)
1AZ–2PF	21/23	(91.3%)	23/23	(100%)	2/2	(100%)	2/2	(100%)	11/23	(47.8%)
1MO–2MO	0/1	(0%)	1/1	(100%)	0/1	(0%)	1/1	(100%)	0/1	(0%)
1PF–2PF	26/34	(76.5%)	34/34	(100%)	8/12	(66.7%)	12/12	(100%)	13/34	(38.2%)
* p*-value	0.106		1		0.233		1		0.743	

*p*-values were calculated by two-sided chi square test with post-hoc analysis.

*AZ* AstraZeneca COVID-19 Vaccine, *ichroma nAb* ichroma™ COVID-19 nAb assay, *MO* Moderna COVID-19 Vaccine, *N/A* not applicable, *PF* Pfizer-BioNTech COVID-19 vaccine, *QFN-SARS* QuantiFERON-TB SARS-CoV-2 ELISA Kit.

**Figure 3:**
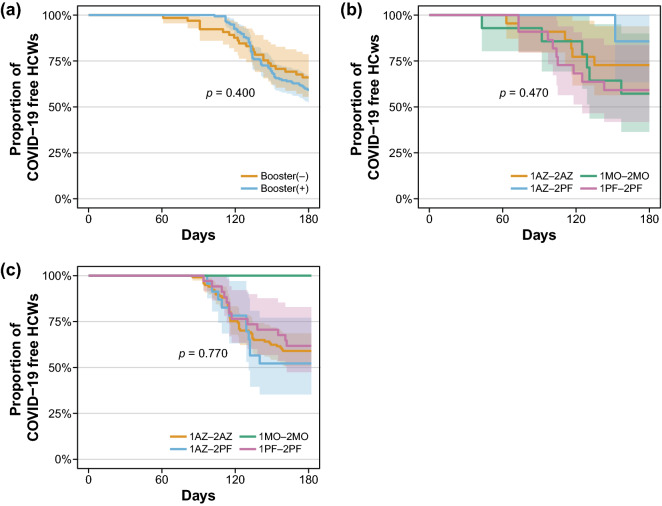
Kaplan–Meier plots of COVID-19-free follow-up depending on the booster shot (**a**), and the type of SARS-CoV-2 vaccination for booster (−) group (**b**) and booster (+) group (**c**).

Similarly, we performed Kaplan–Meier analyses to evaluate the protection against SARS-CoV-2 after COVID-19 vaccination (Fig. 3). Comparing the booster (−) and booster (+) groups revealed no difference in the proportion of COVID-19-free HCWs. In addition, there was no association between the type of first and second vaccinations and COVID-19 infection in both the booster (−) and booster (+) groups.

## Discussion

Since the rapid spread of COVID-19 globally, there has been growing public interest in the laboratory tests used for the evaluation of the immune status of individuals with respect to SARS-CoV-2 infection^11,12^. With these global demands, various kits of IGRA-format tests and nAb assays have been introduced in the market for the evaluation of humoral and T-cell-mediated responses^5,13,14^. Although many studies were conducted to confirm the usefulness of these test results^5–7,9,15,16^, there is still insufficient evidence that these test results are clinically useful^13^. Therefore, we aimed to determine the changes in these test results before and after COVID-19 booster vaccination, and with respect to the type of COVID-19 vaccination, to confirm whether these test results can be used to predict the risk of SARS-CoV-2 infection over a long-term follow-up period.

Overall, we enrolled a total of 240 HCW participants during the study period, and 65 participants were tested with 1st QFN-SARS and nAb tests before the booster vaccination (the booster (−) group). Although 84.6% of these booster (−) HCWs were positive for the nAb assay (55/65), only about half of them were positive in the QFN-SARS test (52.3%, 34/65). Within 1 month after the vaccination with the booster shot, the positivities of the QFN-IGRA and nAb tests (1st tests of booster (+) group) rose sharply compared to the booster (−) group. Approximately 3 months after the booster shot (2nd tests of the booster (+) group), the positivity of QFN-SARS decreased to 52.8% (28/53), whereas that of nAb remained high (100%, 53/53). Similar trends were observed in the Kaplan–Meier analysis with a linear decreasing trend of the positive rate of QFN-SARS with respect to the number of days after the last vaccination, while that of the nAb assay was over 80% until 6 months after the last vaccination. These results indicate that the antibody response caused by the SARS-CoV-2 vaccination lasts relatively long, although the response of the T-cells disappears quickly.

Recently, Murugesan et al. investigated the long-term accuracy of the SARS-CoV-2 IGRA assay and anti-SARS-CoV-2 IgG antibody assays^12^. At 0.5 month post infection, 100% and 87.5% of the participants tested positive in the IGRA-SARS and IgG assays; however, the positive rates of both assays decreased to 79.5% and 71.8% at 10 months after COVID-19 infection, respectively. For both assays, the decline in positive rates seemed to be slower than the results of our study; these findings would indicate that the duration of actual infection-induced immunity is longer than that of vaccine-mediated immunity.

With the 1st test results of QFN-SARS and ichroma nAb, we attempted to analyze the relationship between these in vitro test results and SARS-CoV-2 infection. There was no significant difference in SARS-CoV-2 infection between the positive and negative groups of the ichroma nAb assay. However, paradoxical results were observed—the proportion of COVID-19-confirmed cases among the QFN-SARS-positive participants was higher than that among the QFN-SARS-negative participants. During the study period, an epidemic caused by the Omicron variant broke out in the Republic of Korea^17,18^. Although we could not consider various factors related to SARS-CoV-2 infection, the results of our study showed that the in vitro IGRA and nAb tests against SARS-CoV-2 could not be used as indicators to predict the risk of COVID-19 infection, especially the Omicron variant.

Considering the test results according to the type of vaccination, only the booster (−) group showed significant differences in the positive rates of QFN-SARS and ichroma nAb. We assumed that the distribution of the number of days after the last vaccination was relatively wide in the booster (−) group, which could have affected the positive rates of QFN-SARS and ichroma nAb. In the booster (+) group, the positive rates of QFN-SARS and ichroma nAb did not differ according to the type of vaccination. These results would indicate that the T-cell-mediated immunity and humoral immunity as measured by these in vitro tests would be affected not by the type of vaccination, but by the elapsed time since the last vaccination. The proportion of COVID-19-confirmed participants was not influenced by the type of vaccination in both the booster (−) and booster (+) groups, which indicates that the type of vaccination is not associated with protection against the Omicron variant infection. Therefore, we would recommend that other factors such as close contacts of infected patients, high viral load exposure, comorbidity, or host immune status should be considered when assessing the risk of COVID-19 infection^19–21^, rather than the type of vaccination and the in vitro test results.

Despite the significant results of this study, there are several limitations to our study. First, although there were no deaths due to SARS-CoV-2 infection, we retrospectively collected only the history of COVID-19 infection through a questionnaire, not the symptoms. Therefore, the correlation between the test results and the symptomatic characteristics of COVID-19 could not be analyzed. In addition, there may be hidden infections that have not been diagnosed due to asymptomatic infection. Second, because the study participants were limited to HCWs, we enrolled only relatively young and healthy persons under the age of 60; thus, the results of this study would not be representative of the entire population. In addition, about two-thirds of the participants did not voluntarily dropped out in the second test, possibly resulting in selection bias. Therefore, further large-scale studies should be conducted to overcome the present limitations and provide more informative results regarding the IGRA-SARS and nAb assays.

Our study provides comprehensive QFN-SARS and ichroma nAb test results that reflect T-cell mediated and humoral immunity against SARS-CoV-2 with respect to the number of days after the last vaccination and the type of vaccination. The positive rates of QFN-SARS and ichroma nAb rapidly increased after booster vaccination, and the positivity of the nAb assay remained high until 6 months after the last vaccination. However, the positivity of QFN-SARS showed a rapid decline, and about half of the participants were negative about 3 months after the last vaccination. Our findings also suggest that these in vitro test results and the type of vaccination may not be useful to predict the risk of COVID-19 infection. The results of our study indicate that although the in vitro tests for measuring immunity against SARS-CoV-2 may reflect SARS-CoV-2 vaccination well, their clinical usefulness is still unclear and further large-scale studies should be performed to address this point.

## Materials and methods

### Study participants

A total of 240 HCWs working in Chung-Ang University Hospital were enrolled in this study. We only enrolled HCWs without any history of COVID-19 infection. All participants were vaccinated twice at the time of enrollment in this study, and only those who wanted booster shots were vaccinated with third vaccination. Based on the booster vaccination, the participants were divided into two groups: booster (−) group and booster (+) group. The first specimens for all participants were sampled at the beginning of this study, and the second blood sampling was done at the end of the study on some subjects of the booster (+) group. At the time of collecting the 1st and 2nd blood specimens, HCWs with a history of COVID-19 infection were excluded. With these specimens, we used the QFN-SARS and the ichroma nAb. At the end of the study in April 2022, we investigated the history of SARS-CoV-2 infection of all the enrolled participants through a questionnaire. The timeline and groups of this study are shown in Supplementary Fig. S1.

This study was conducted from November 2021 to April 2022 at the Chung-Ang University Hospital (Seoul, Republic of Korea), and blood samples were drawn after informed consent was obtained from all the participants.

### QuantiFERON-TB SARS-CoV-2 ELISA

The QFN-SARS is a qualitative assay for the detection of IFN-γ released in response to in vitro stimulation by a specific SARS-CoV-2 antigen in human whole blood. For QFN-SARS, 4 ml of whole blood was collected into four QuantiFERON blood collection tubes, which included a Nil tube (negative control), a mitogen tube (positive control), and two S peptide-containing tubes with SARS CoV-2 (Ag1 and Ag2) to stimulate the immune cells. These tubes were gently shaken to ensure adequate mixing of the additive and blood and transferred to a 37 °C incubator. After 16–24 h of incubation, the tubes were centrifuged at 2500×*g* for 15 min and the plasma separated. Then, levels of IFN-γ were measured for the plasma samples by ELISA. The QFN-SARS results were interpreted according to the manufacturer’s specifications (Supplementary Table S1).

### ichroma™ COVID-19 nAb assay

ichroma nAb is a qualitative fluorescence immunoassay for the detection of neutralizing antibodies against SARS-CoV-2. The target antibody of this test is the neutralizing antibody that block the interaction between the receptor binding domain (RBD) of the viral spike glycoprotein with the ACE-2 cell surface receptor. To evaluate the neutralizing antibody status, whole blood samples were drawn, and the serum was separated within 3 h after the collection of whole blood. During the test procedure, the SARS-CoV-2-neutralizing antibody in the sample binds to the fluorescence-labeled SARS-CoV-2 Spike RBD antigen to form the antigen–antibody complex. Then, this complex is loaded onto lateral flow immunoassay kit, where the covalent couple of ACE2 is immobilized, and interferes with the binding of analyte and Fluorescence-labeled (FL) antigen. With the increasing presence of the analytes in the blood specimens, more of the fluorescent antigen is bound, resulting in a lower fluorescence signal. The fluorescence signal was quantified using ichroma™ II (Boditech Med) which is a compact, fluorescence-based immunoassay analyzer, and the test result was reported as ‘Positive’ or ‘Negative’ based on the cut-off index.

### Ethical statement

This study was performed in accordance with the relevant guidelines and regulations, and approved by the institutional review board of Chung-Ang University Hospital (IRB No. 2108-007-473). All participants provided written informed consent.

### Statistics

All statistical analyses were performed using R version 4.3.3 (http://www.R-project.org/). Median values were calculated with interquartile ranges for quantitative variables. Qualitative variables were expressed as percentages (%), and a two-sided chi square test with post-hoc analysis was used to compare qualitative variables. We used the Kaplan–Meier method to analyze the time-to-event data (the change of test results and SARS-CoV-2 infection). *p* value < 0.05 was considered statistically significant.

## Supplementary Information

Supplementary Information.

## Data Availability

The datasets generated during the current study are available from the corresponding author on reasonable request.
