# Supplementary material for: Exploring the vaccine-induced immunity against severe acute respiratory syndrome coronavirus 2 in healthcare workers
Source: Sci Rep. 2023 Apr 26;13:6830. doi: 10.1038/s41598-023-33397-4 (PMC10131514; doi:10.1038/s41598-023-33397-4)
Supplement: Supplementary file 1 — Supplementary Information. [file 41598_2023_33397_MOESM1_ESM.docx]

**Supplementary Information**

**Exploring the Vaccine-induced Immunity against Severe Acute Respiratory Syndrome Coronavirus 2 in Healthcare Workers**

Yong Kwan Lim, M.D.^1^, Oh Joo Kweon, M.D.^1^, Yoojeong Choi^1^, Sumi Yoon, M.D.^1^, Tae-Hyoung Kim, M.D.^2^, Mi-Kyung Lee, M.D.^1,*^

^1^Department of Laboratory Medicine, Chung-Ang University College of Medicine, Seoul, Republic of Korea

^2^Department of Urology, Chung-Ang University College of Medicine, Seoul, Republic of Korea

*Corresponding author, email: cpworld@cau.ac.kr

**
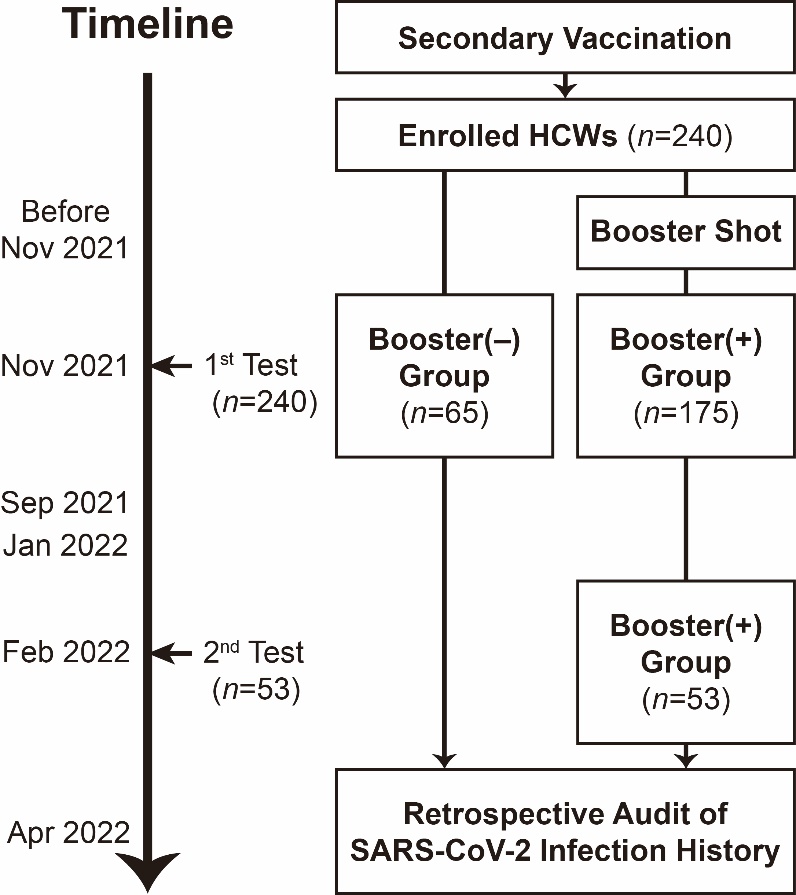
**

**Supplementary Figure S1.** Timeline and flowchart of study. All enrolled healthcare workers (HCWs) were categorized into booster(-) and booster(+) groups based on booster vaccination.

**Supplementary Table S1.** Interpretation criteria of QFN-SARS test results

| Nil (IU/ml) | AG1 minus Nil (IU/ml) | AG2 minus Nil (IU/ml) | MIT minus Nil (IU/ml) | Result |
| --- | --- | --- | --- | --- |
| ≤8.0 | <0.15 or ≥0.15 and <25% Nil | <0.15 or ≥0.15 and <25% Nil | ≥0.50 | Non-reactive |
| ≤8.0 | ≥0.15 and ≥25% Nil | Any | Any | Reactive |
| ≤8.0 | Any | ≥0.15 and ≥25% Nil | Any | Reactive |
| ≤8.0 | <0.15 or ≥0.15 and <25% Nil | <0.15 or ≥0.15 and <25% Nil | <0.50 | Indeterminate |
| ≥8.0 | Any | Any | Any | Indeterminate |

The plasma levels of IFN-γ for the Ag1, Ag2, Nil, and mitogen tubes are referred to as TB1, TB2, Nil, and MIT, respectively.
